# Balanced Input Allows Optimal Encoding in a Stochastic Binary Neural Network Model: An Analytical Study

**DOI:** 10.1371/journal.pone.0030723

**Published:** 2012-02-16

**Authors:** Gustavo Deco, Etienne Hugues

**Affiliations:** 1 Center of Brain and Cognition, Universitat Pompeu Fabra, Barcelona, Spain; 2 Institució Catalana de Recerca i Estudis Avançats (ICREA), Universitat Pompeu Fabra, Barcelona, Spain; Georgia State University, United States of America

## Abstract

Recent neurophysiological experiments have demonstrated a remarkable effect of attention on the underlying neural activity that suggests for the first time that information encoding is indeed actively influenced by attention. Single cell recordings show that attention reduces both the neural variability and correlations in the attended condition with respect to the non-attended one. This reduction of variability and redundancy enhances the information associated with the detection and further processing of the attended stimulus. Beyond the attentional paradigm, the local activity in a neural circuit can be modulated in a number of ways, leading to the general question of understanding how the activity of such circuits is sensitive to these relatively small modulations. Here, using an analytically tractable neural network model, we demonstrate how this enhancement of information emerges when excitatory and inhibitory synaptic currents are balanced. In particular, we show that the network encoding sensitivity -as measured by the Fisher information- is maximized at the exact balance. Furthermore, we find a similar result for a more realistic spiking neural network model. As the regime of balanced inputs has been experimentally observed, these results suggest that this regime is functionally important from an information encoding standpoint.

## Introduction

Cognitive behavior requires an efficient selection of relevant information from the enormous amount of sensory information continuously flowing into the brain. The perceptual system performs this selective filtering process by relying on attentional mechanisms by which a behaviorally relevant stimulus in the environment is enhanced relative to other irrelevant distractors. During the last years, many experiments have found as neuronal correlates of attention a modulation of the firing rate activity (see e.g. [1]–[5]). More recently, experiments have shown that attention affects the neural variability –as measured by the Fano factor- and correlations over trials [6]–[8]. In these experiments, single cells in V4 were recorded in awake behaving monkeys when one stimulus in the neuron's receptive field was behaviorally attended or non-attended. Both studies reported a relatively small but significant decrease of both the Fano factor (mean-normalized variance of the neural spike counts over trials) and neuronal correlations in the attended condition with respect to the non-attended one. Additionally, several studies across a variety of species, cortical areas, brain states and stimulus conditions have found that stimulus onset generally reduces neural variability [9]. These attention induced reductions suggest an enhancement of the information necessary to select and further process the relevant stimulus. Indeed, these reductions improve the signal-to-noise ratio and eliminate redundancy, both crucial features for enhancing the encoding of information.

As attention just modulates neural activity, it is believed to be conveyed to a given neural circuit by a relatively small signal. Beyond the attentional paradigm, which is well suited for experimental investigation, the local activity in a neural circuit can be modulated in a number of ways. Therefore, a general question is to understand how the activity of such circuits depends on such modulations or, in other words, how sensitive the activity is.

In this paper we study, first for an analytically tractable model and then with simulations of a biophysical model, the conditions under which a neuronal network encodes information with maximal sensitivity. We study the encoding sensitivity from an information-theoretical point of view by using the Fisher information. We demonstrate analytically that the encoding sensitivity is maximized in the balanced input regime and confirm this result for the biophysical model. The balanced input regime is supported by experimental observations in vitro [10] and in vivo [11], [12]. In turn, we find that the variability also maximally decreases around the balance. In conclusion, the present results suggest that the balanced regime allows the best encoding sensitivity.

## Results

### An Analytically Solvable Stochastic Binary Neural Network Model

To illustrate this basic phenomenon we will present an analytically tractable neural network model, a network of stochastic binary neurons –or an Ising spin model in statistical physics [13], which allows to derive analytically the variability, measured by the Fano factor, and the sensitivity to an external stimulation, measured by the Fisher information. We then demonstrate that the sensitivity to an external information is maximal in the regime where there is a balance between excitatory and inhibitory afferent inputs. Then, we show that this theoretical prediction extends to the case of a biophysically realistic neural network model of spiking neurons with AMPA, NMDA and GABA synapses.

A stochastic binary neuron takes the output value 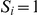 with probability 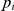 and the value 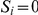 with probability 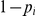. The probability 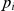 is given by
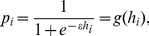
(1)where 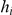 denotes the total input to this neuron. In such neurons, the stochasticity is modeled by “thermal” fluctuations (the parameter 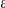 in Equation 1 denoting an inverse temperature), a convenient way to represent the influence of noise on this neuron. In statistical physics, defining such probabilities defines a Glauber dynamics [14], which provides a way to calculate the evolution of the network state. Note that, even if our present results do not necessitate the explicit use of the Glauber dynamics, this evolution should consider asynchronous updating of the neurons.

**Figure 1:**
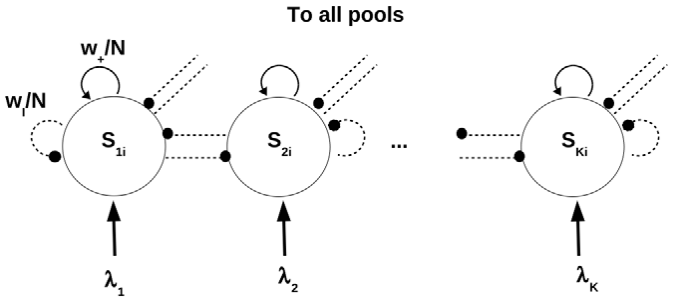
Architecture of the stochastic binary neurons network with Glauber dynamics. (See text for details).

The network we consider (see Figure 1) consists of 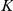 mutually and recurrently inhibiting populations of 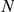 neurons with weigth 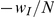. Each population is also recurrently connected with excitatory weigth 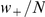, 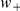 being denoted the cohesion level. The network is fully connected. The population 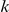 receives the external stimulation 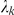. As a special case, we will consider that population 1 encodes a target stimulation, the input comprising and external stimulation 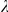 plus a top-down extra input 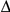 (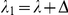), whereas all other populations encode distractors and receive only the stimulation 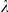
(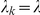, 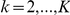).

Let us denote by 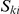 the state of the neuron 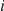 in population 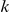. The Glauber dynamics of this network can be described by the following equations
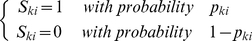
(2)where 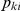 is given by
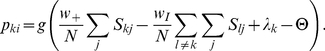
(3)Note that, due to the connectivity considered here, these probabilities are identical across each population. Let us further label a given configuration of all the 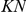 neurons by a superindex 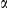. For a symmetric connectivity -as here, at large times, the probability of finding the system in a specific state 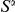 is given by the Boltzmann-Gibbs distribution
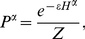
(4)where 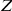 is the partition function defined by
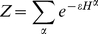
(5)and 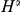 is the energy function –of the configuration 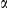
- given by
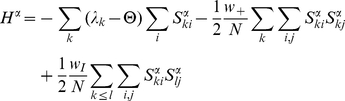
(6)The averaged mean activity in each population 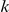 is defined by:
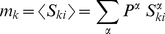
(7)Deriving the mean-field approximation (see [13]), we can write the fixed point equations describing the averaged mean population activity as
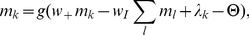
(8)for 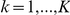. For a given set of parameters 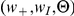, and inputs 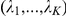, these 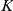 nonlinear equations determine the 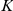 unknowns 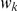.

The averaged second moment of a population can be shown to be given by
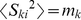
(9)and consequently the Fano factor, defined as the variance over the mean of the population activity, is
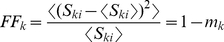
(10)To characterize the encoding sensitivity of the network, we use the Fisher information. This information theoretic quantity describes the amount of information that an observable random variable 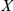 carries about a given parameter upon which the probability of 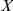 depends. Moreover, for any unbiased estimator of this parameter, its variance is always greater than the inverse of the Fisher information, a lower bound called the Cramer-Rao bound. Therefore, the higher the Fisher information is, the better one can estimate the given parameter from the observation of the variable 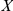. Here, we will study how sensitively the network encodes 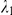. For a network of stochastic neurons whose distribution of states follows the Boltzmann-Gibbs distribution, the Fisher information, defined by
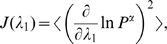
(11)can be rewritten
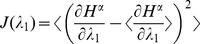
(12)which, after some straightforward algebra, is expressed by
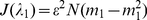
(13)The maximum of the Fisher information with respect to 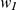 is given by the condition:
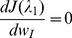
(14)Inserting Equation 13 into Equation 14, the possible solutions are 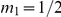 and 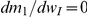. However, the second solution is possible only for high cohesion level 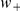, a case we do not consider here. Using Equation 8, we finally find:
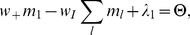
(15)which means that the Fisher information is maximal when the excitatory and inhibitory synaptic currents are exactly balanced in the first population.

**Figure 2:**
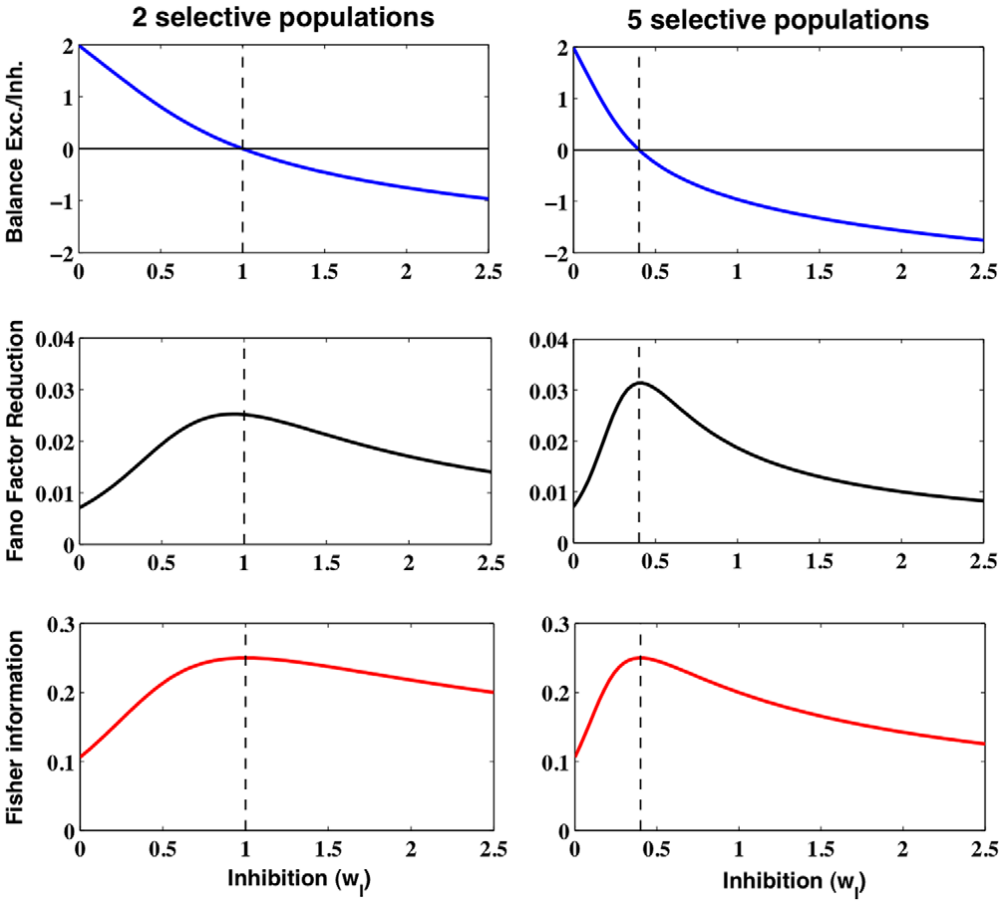
Stochastic binary neurons network behavior as a function of the inhibition level 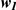
 **.** Current balance (top), Fano factor reduction (middle) (for a bias 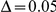) and Fisher information scaled down by 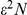 (for a bias 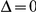) (bottom). (**Left**) Case of 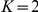 selective populations, for which input balance occurs at 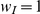 (dashed line). (**Right**) Case of 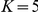 selective populations, for which input balance occurs at 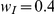 (dashed line). All results are analytical.

In the following, and in our simulations, we have considered the simpler symmetric input 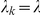, adding a bias 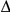 to 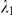 (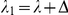) to consider the effect of a top-down input, or an attentional input when the stimulus to which the first population is selective is attended. The symmetric input case is interesting because this is the extreme case where the network sensitivity to a bias can be studied while all selective populations have the same activity. We used the following parameter values: 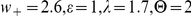. When 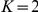, the balanced competition occurs for 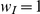, and for 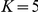 it occurs for 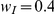 (see Equation 15). Note that, for these parameters, the fixed point obtained from the system of Equations 8 is symmetrical (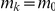 for all 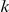) when 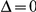. Figure 2 shows the balance condition, the Fano factor reduction (for 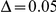) and the Fisher information (for 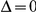 and scaled down by 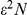) as a function of the inhibition level for both values of 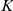. We note that the Fano factor reduction peaks around the point where the input is balanced. In Appendix S1, we demonstrate analytically that the Fano factor reduction peaks around the point where the Fisher information peaks. In conclusion, using a network of stochastic neurons, we were able to demonstrate analytically that the maximal sensitivity, as measured by the Fisher information, occurs exactly when the excitatory and inhibitory synaptic currents are balanced.

### A Biophysically Realistic Spiking Neural Network Model

We now study here a biophysically realistic neural network model [15]. The model uses integrate-and-fire neurons with excitatory (AMPA and NMDA) and inhibitory (GABA-A) synaptic receptor types. It is formulated and analyzed in the theoretical framework of attractor networks introduced in the seminal work of Amit [16]. An attractor network is a neural network whose dynamical state has the tendency to settle into a stable firing pattern, which eventually destabilizes under the effect of noise. Its behavior can be formally described by dynamical systems theory (see Materials and Methods).

As in the previous model, the network has 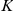 selective excitatory neural pools. We consider the case where 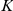 stimuli are presented simultaneously and when an external bias is applied to the first population. For attention, this bias models the attentional signal that this population receives when the corresponding stimulus is attended, as suggested by previous studies [3], [17]. For 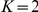, this corresponds to the case where 2 stimuli are presented simultaneously in a neuron's receptive field and when attention is allocated to only one of them, this one being the target and the other one the distractor. This situation is referred to lead to “biased competition” [1], [2]. Each simulation started with a period of 500 ms (for network activity stabilization), followed by a period of 1000 ms where an identical stimulus was presented to all selective populations, represented by the corresponding extra rates 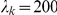
Hz,. As before, in this network state, we are interested in the sensitivity of the network activity to a small modulatory input -or bias- applied to population 1, and implemented here by adding an extra input rate 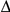 to this population (i.e. 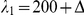
Hz). We have considered both the case of a very small bias (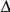 almost 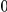) and the case of of 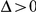 but small. The spiking activity was averaged over 4000 trials, initialized with different random seeds.

**Figure 3:**
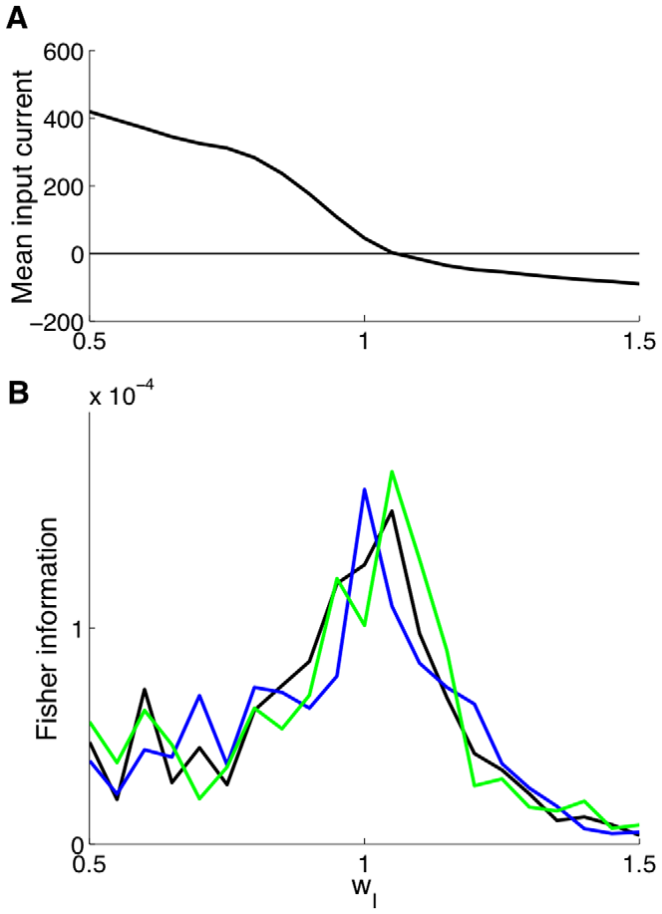
Spiking neurons network behavior as a function of the inhibition level 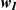
 **.** (**A**) Mean synaptic current and (**B**) estimated Fisher information for the population receiving an extra bias 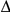. This quantity measures the network activity sensitivity to the bias 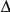 and is calculated at bias 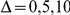 Hz (black, blue and green curves, respectively). The Fisher information peaks around the excitatory and inhibitory synaptic currents balance. Due to noise in the data, it is almost impossible to distinguish the different curves.

Again, the sensitivity of the network activity to the external bias 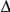 is evaluated using the Fisher information. We estimate this quantity numerically with respect to the underlying synaptic currents balance, and right from its definition, involving the spike count distribution (see Materials and Methods). Figure 3B shows, for several values of the bias 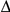, its evolution as a function of the inhibition level. Comparing with the mean input current to which is subtracted the threshold current (see Figure 3A), the Fisher information clearly peaks around input balance, which occurs around 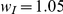. For lower inhibition levels, the Fisher information tends to a plateau, and for higher ones, it tends to zero, as the network tends to silence. Although we took quite large values of the bias (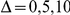 Hz) and a large number of trials, noise in the data still prevents to distinguish Fisher information for different values of the bias 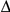.

**Figure 4:**
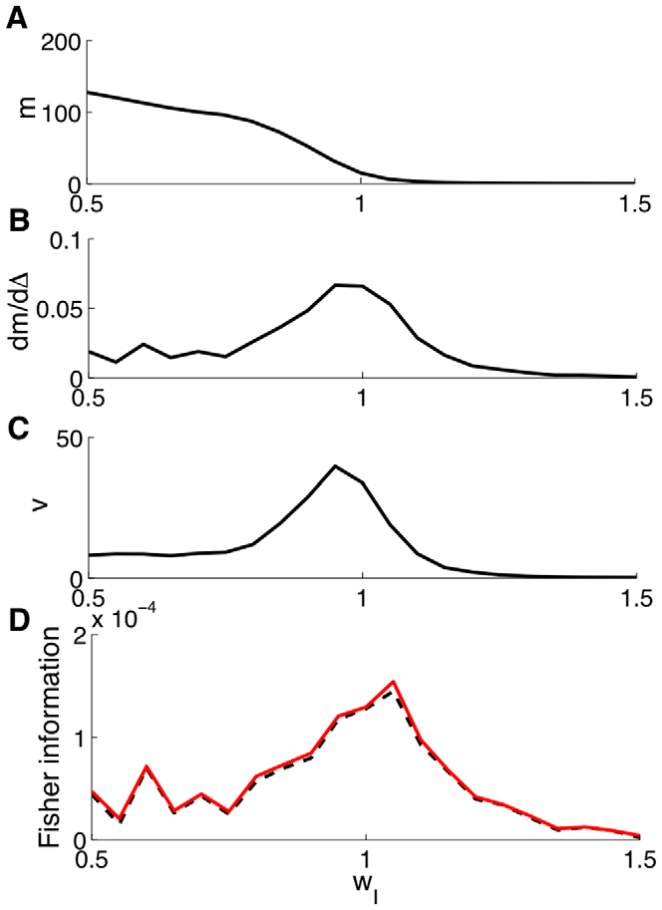
Fisher information behavior for the spiking neurons network as a function of the inhibition level 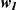
 **.** (**A**) Mean spike count 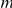, (**B**) its derivative with respect to the bias, 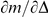, (**C**) the spike count variance 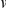 and (**D**) the estimated Fisher information (red) and its analytical fit 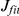 (dashed black). The analytical fit works very well, showing that the Fisher information peaks around input balance because 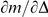 also peaks there. In turn, this quantity peaks because the mean spike count 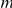 has, with respect to 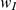, a maximum (absolute) slope around balance.

To further investigate the behavior of the Fisher information, we have derived an approximate analytical expression (see Appendix S2). First, for high inhibition values, neurons receive a subthreshold current (see Figure 3A), and their firing tends to become Poissonian. In this regime, we evaluate analytically the Fisher information from the Poisson spike count distribution. Second, for low inhibition values, neurons have a current well above the threshold (see Figure 3A), and the spike count distribution can be well fitted by a Gaussian, whose mean is given by the mean spike count 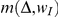 and variance by 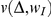. The Fisher information is also evaluated analytically in this case. Finally, it can be shown that these two formulas have the same form (see Appendix S2), namely
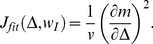
(16)In Figure 4 we plot, with respect to 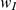, 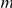, 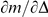, 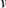 and compare the estimated Fisher information with 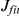, which is shown to fit very well for all values of the inhibition level 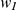. Finally, the Fisher information is found to peak because 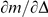 peaks around input balance. In turn, this quantity peaks because the mean spike count 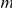 has, with respect to 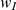, a maximum (absolute) slope around balance.

## Discussion

How attention increases the encoding of the information necessary for the selection of the relevant stimulus? More generally, how the activity in a local neural circuit changes in response to a modulatory input? Here, using an analytically tractable model, we rigorously investigated under which conditions this modulation is better detected. Using an information-theoretic measure, the Fisher information, we were able to show that the maximum sensitivity of the system occurs when the excitatory and inhibitory synaptic currents are balanced. Note that logically, but not trivially, this maximum also corresponds to the maximum slope of the single neuron response function. Furthermore, we found a similar conclusion using a more realistic model.

The balanced input regime has received quite a lot of experimental and theoretical support. Experimental observations in vitro [10] and in vivo [11], [12] have revealed that the ratio of excitatory and inhibitory input conductances remains remarkably stable over time, within and across neurons in active local networks, either in a balanced way or favoring inhibition. On the theoretical side, using statistical physics tools, Buice and Cowan [18] have shown how balanced excitation and inhibition and criticality are related. Furthermore, they analyze the advantage of having a system of spiking neurons at criticality and present numerous empirical evidences of cortical systems working at this type of critical point. Essentially, they discuss the fact that, at this critical balanced state, the system is mainly driven by fluctuations and therefore variability and correlations are much more sensitive to external influences, conclusions which are consistent with ours. Other theoretical studies [19], [15], [20] have indicated that the balance input regime is convenient to sustain a stable spontaneous state, and allows rapid transitions between relatively stable network states, which can modulate the neural responsiveness in a behaviorally relevant manner. One classical example is attention: a balanced network is suitable to mediate biased competition [4], [3], i.e. it is particularly able to amplify the rate modulations induced by modest external bottom-up or top-down attentional biases.

## Materials and Methods

The biophysically realistic model uses integrate-and-fire neurons with excitatory (AMPA and NMDA) and inhibitory (GABA-A) synaptic receptor types.

### Neurons and Synapses

The spiking activity of neurons in the network is described by an integrate-and-fire model. Integrate-and-fire (IF) neurons are point-like elements, whose dynamical state is described by their membrane potential 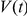. An IF neuron can be described by a basic circuit consisting of a cell membrane capacitance 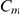 in parallel with a membrane resistance 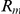, driven by input currents coming from connected neurons. Hence, the subthreshold dynamics of the membrane potential of each neuron in the network is given by the following equation:
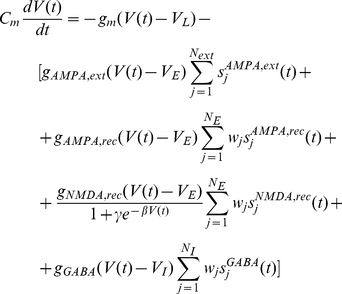
(17)where 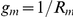 is the membrane leak conductance, 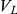 is the resting potential, and 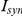 is the synaptic current. The membrane time constant is defined by 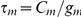. When the voltage across the membrane reaches a given threshold 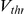, the neuron generates a spike which is then transmitted to other neurons and the membrane potential is instantaneously reset to 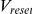 and maintained there for a refractory time 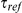 during which the neuron is unable to produce further spikes. The spikes arriving to a given neural synapse produce an input to the neuron which induce post-synaptic excitatory or inhibitory potentials (through a low-pass filter formed by the membrane and synaptic time constants). In Equation 17, 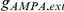, 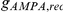, 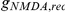, and 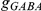 are the synaptic conductances, and 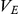, 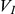 the excitatory and inhibitory reversal potentials, respectively. The dimensionless parameters 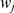 of the connections are the synaptic weights. The NMDA currents are voltage dependent and they are modulated by intracellular magnesium concentration. The gating variables 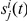
are the fractions of open channels of neurons and they are given by:
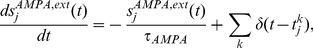
(18)

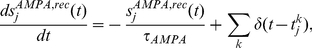
(19)

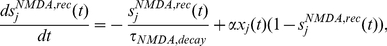
(20)

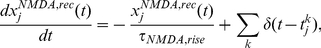
(21)

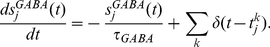
(22)The sums over the index 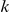 represent all the spikes emitted by the presynaptic neuron j (at times 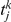). In Equations 18–22, 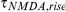 and 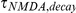 are the rise and decays times for the NMDA synapses, and 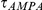 and 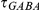 the decay times for AMPA and GABA synapses. The rise times of both AMPA and GABA synaptic currents are neglected because they are short (<1 ms). The values of the constant parameters and default values of the free parameters used in the simulations are displayed in Table 1.

**Table 1:** Neural and synaptic parameters.

Excitatory neurons		Inhibitory neurons		Synapses	
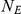	800 neurons	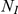	200 neurons	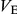	0 mV
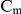	0.5 nF	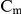	0.2 nF	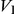	−70 mV
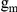	25 nS	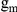	20 nS	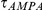	2 ms
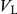	−70 mV	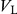	−70 mV	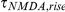	2 ms
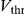	−50 mV	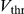	−50 mV	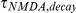	100 ms
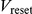	−55 mV	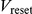	−55 mV	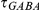	10 ms
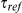	1 ms	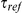	1 ms	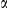	0.5 ms^−1^
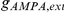	2.08 nS	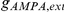	1.62 nS	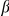	0.062 mV^−1^
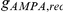	0.104 nS	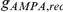	0.081 nS	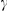	0.2801
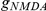	0.327 nS	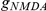	0.258 nS	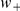	1.9
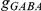	1.25 nS	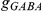	0.973 nS		

Parameter values for the spiking neural network model. In the numerical simulations, the parameters entering in the definition of neuron and synaptic models take the given values (See Materials and Methods).

### Neural Network

The network consists of 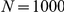 interacting neurons, where 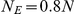 are excitatory (pyramidal) cells and 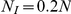 are inhibitory cells (interneurons), consistent with the neurophysiologically observed proportions [21]. We use an attractor network where neurons are organized into a discrete set of populations. There are three different types of populations, namely: 1) the inhibitory population, 2) the excitatory non-selective populations and 3) the excitatory selective populations. The inhibitory population is made of the inhibitory neurons in the modeled brain area and mediates competition in the attractor network by distributing a global inhibitory signal. The non-selective population 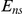 is composed of all excitatory neurons that are not receiving any specific external input and which therefore provide a background level of excitation. The remaining excitatory neurons are clustered in 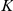 different specific populations 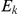 (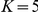 in the present simulations). Each contains 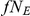 neurons (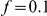 in the present simulations) which are sensitive to a specific external stimulus. The network is fully connected, meaning that each neuron in the network receives 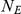 excitatory and 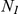 inhibitory synaptic contacts. The connections strengths between and within the populations are determined by dimensionless weights 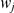. We assume that the connections are already formed, e.g. by earlier self-organization mechanisms, as if they were established by Hebbian learning, with the coupling between two neurons being strong if their activities are correlated and weak if they are anticorrelated. The recurrent self-excitation within each selective population 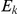 is given by the weight 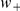 (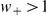), which is called the cohesion level, and the weaker connection between them by the weight 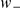
(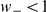). The synaptic efficacy 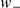 depends on 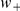 by the relation 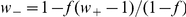: this relation ensures that the average excitatory synaptic efficacy remains constant when 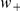 varies. Neurons in the inhibitory population are mutually connected with an intermediate weight 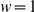. These neurons are also connected with all excitatory neurons with the same intermediate weight, which for excitatory-to-inhibitory connections is 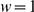 and, for inhibitory-to-excitatory connections is denoted 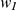, called the inhibition level. Neurons in each excitatory population 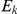 are connected to neurons in the population 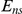 with a feedforward and feedback synaptic weights 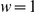 and 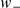, respectively. The remaining connections are all set to the baseline value, i.e. to 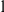.

All neurons in the network always receive an external background input from 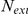 external neurons emitting uncorrelated Poisson spike trains at rate 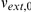. The resulting spike train is still a Poisson spike train, with rate 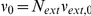. More specifically, and for all neurons inside a given population p, the resulting spike train is assumed to have a time-varying rate 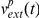, governed by
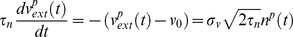
(23)where 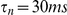, 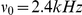, 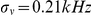 is the standard deviation of 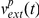 and 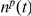 is a normalized Gaussian white noise. Due to noise, negative values of 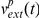 that could arise are rectified to zero. These input rate fluctuations represent the noisy fluctuations that are typically observed *in vivo*. Additionally, neurons in a specific selective population 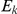 receive other inputs when an external stimulus is applied (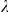) or a bias (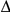) to that population. These inputs are specified by adding a corresponding rate to the rate of the background Poissonian input spike train.

### Fisher Information

For the spiking network simulations, to measure how sensitively the activity of one population receiving a stimulus with an extra bias can be detected, we calculated the amount of information that the neural spike counts (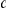) in this population carry about the bias 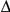. To evaluate the distribution 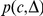 for a given 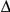, we use the spike counts for a time window of 500 ms for the 80 neurons in the attended population and over 4000 trials. We estimated numerically the Fisher information from its definition
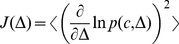
(24)by calculating first the empirical distributions 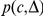 for different values of 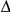, and calculating the derivative with respect to 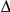 using a centered discretization formula (we used a 10 Hz discretization step to ensure enough precision). In Figure 4, we evaluate numerically the Fisher information as a function of 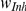 in the limit of infinitesimal bias (i.e. 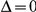 Hz), the sensitivity being non-zero in this limit, and for positive values (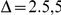 Hz).

## Supporting Information

Appendix S1 **For the stochastic binary neurons network, we demonstrate analytically in this appendix why the Fano factor reduction is maximum around the same value that the Fisher information is maximum, which is for a balanced input.**
(DOC)

Appendix S2 **To better understand the behavior of the Fisher information with respect to parameters, we derive in this appendix approximate analytical expressions.**
(DOC)
