# Supplementary material for: Balanced Input Allows Optimal Encoding in a Stochastic Binary Neural Network Model: An Analytical Study
Source: PLoS One. 2012 Feb 16;7(2):e30723. doi: 10.1371/journal.pone.0030723 (PMC3281140; doi:10.1371/journal.pone.0030723)
Supplement: Appendix S1 — For the stochastic binary neurons network, we demonstrate analytically in this appendix why the Fano factor reduction is maximum around the same value that the Fisher information is maximum, which is for a balanced input. (DOC) [file pone.0030723.s001.doc]

**Appendix S1**

While the Fisher information is maximum for a balanced input, we have observed (see Fig. 2) that the maximum of the Fano factor reduction occurs close to balance. In this appendix, we show why this is so. As we have considered small values of the bias, in the general input case, the Fano factor reduction for the pool $k$ can approximately be written as

$-\frac{\partial FF_{k}}{\partial\lambda_{1}}=\frac{\partial m_{k}}{\partial\lambda_{1}}$ (A1)

where the right hand side, noted below $m_{k}^{'}$, comes from Equation 10. Deriving with respect to $\lambda_{1}$ the set of Equations 8 $m_{k}=g(h_{k})$, we get the following system of linear equations

$\left\{\begin{matrix} m_{1}^{'}=\left[w_{+}m_{1}^{'}-w_{I}\sum_{l} m_{l}^{'}+1\right]g_{1}^{'} \\ m_{k}^{'}=\left[w_{+}m_{k}^{'}-w_{I}\sum_{l} m_{l}^{'}\right]g_{k}^{'} \end{matrix}\right.$ (A2)

where the last equations are for $k>1$ and where $g_{k}^{'}=\frac{dg}{dh}(h_{k})$. Rewriting this system in matrix form

$A\left(\begin{matrix} m_{1}^{'} \\ .. \\ .. \\ .. \\ m_{K}^{'} \end{matrix}\right)=g_{1}^{'}\left(\begin{matrix} 1 \\ 0 \\ .. \\ .. \\ 0 \end{matrix}\right)$ (A3)

where $A$ is the matrix of coefficients, we easily deduce that $m_{k}^{'}=g_{1}^{'}\left(A^{-1}\right)_{k1}$. This means that the Fano factor reduction for any pool is proportional to $g_{1}^{'}=\varepsilon m_{1}(1-m_{1})$ which is, up to a multiplicative constant, the Fisher information $J(\lambda_{1})$. As this last quantity peaks when the input is balanced, this is logical that the Fano factor reduction peaks almost at balance.
