# Supplementary material for: Balanced Input Allows Optimal Encoding in a Stochastic Binary Neural Network Model: An Analytical Study
Source: PLoS One. 2012 Feb 16;7(2):e30723. doi: 10.1371/journal.pone.0030723 (PMC3281140; doi:10.1371/journal.pone.0030723)
Supplement: Appendix S2 — To better understand the behavior of the Fisher information with respect to parameters, we derive in this appendix approximate analytical expressions. (DOC) [file pone.0030723.s002.doc]

**Appendix S2**

To better understand the behavior of the Fisher information, we derive here approximate analytical expressions. First, for an inhibition level $w_{I}$ above $1$, neurons receive a subthreshold current (see Figure 3A), and their firing statistics is close to Poisson. The Poisson spike count distribution is

$p(c,\Delta)=\frac{(rT)^{c}}{c!}e^{-rT}$, (B1)

where $r(\Delta,w_{I})$ is the firing rate of the neuron and $T$ the time window duration. If we plug it into the Fisher information expression (see Equation 24), it can easily be found

$J_{P}(\Delta)=\frac{T}{r}\left(\frac{\partial r}{\partial\Delta}\right)^{2}$. (B2)

Second, for an inhibition level $w_{I}$ below $1$, neurons have a current above the threshold (see Figure 3A), and we found that the spike count distribution can be well fitted by a Gaussian (data not shown), whose mean is given by the spike count mean $m(\Delta,w_{I})$ and variance $v(\Delta,w_{I})$. Plugging this Gaussian spike count distribution into the Fisher information expression, it can also easily be found

$J_{G}(\Delta)=\frac{1}{v}\left(\frac{\partial m}{\partial\Delta}\right)^{2}+\frac{1}{2}\left(\frac{1}{v}\frac{\partial v}{\partial\Delta}\right)^{2}$. (B3)

However, for these values of $w_{I}$, the variance is almost constant, eliminating then the second term.

Going back to Equation B2, and writing $m=v=rT$ for a Poisson spike train (for which the Fano factor $v/m=1$), it can finally be shown that both Equations B2 and B3 can be written in the same form

$J_{fit}(\Delta,w_{I})=\frac{1}{v}\left(\frac{\partial m}{\partial\Delta}\right)^{2}$. (B4)

From Figure 4, it can be seen that this expression fits very well the estimated Fisher information for all values of the inhibition level $w_{I}$.
